# MicroRNAs as monitoring markers for right-sided heart failure and congestive hepatopathy

**DOI:** 10.25122/jml-2021-0071

**Published:** 2021

**Authors:** Ruxandra Florentina Ionescu, Sanda Maria Cretoiu

**Affiliations:** 1. Department of Cardiology I, Central Military Emergency University Hospital Dr. Carol Davila, Bucharest, Romania; 2. Department of Morphological Sciences, Cell and Molecular Biology and Histology, Carol Davila University of Medicine and Pharmacy, Bucharest, Romania

**Keywords:** miRNAs, microRNAs, heart failure, cardiac insufficiency, heart remodeling, hepatomegaly, congestive hepatopathy

## Abstract

The last decades showed a worrying increase in the evolution of cardiovascular diseases towards different stages of heart failure (HF), as a stigma of the western lifestyle. MicroRNAs (miRNAs), non-coding RNAs, which are approximately 22-nucleotide long, were shown to regulate gene expression at the post-transcriptional level and play a role in the pathogenesis and progression of HF. miRNAs research is of high interest nowadays, as these molecules display mechanisms of action that can influence the course of evolution of common chronic diseases, including HF. The potential of post-transcriptional regulation by miRNAs concerning the diagnosis, management, and therapy for HF represents a new promising approach in the accurate assessment of cardiovascular diseases. This review aims to assess the current knowledge of miRNAs in cardiovascular diseases, especially right-sided heart failure and hepatomegaly. Moreover, attention is focused on their role as potential molecular biomarkers and more promising aspects involving miRNAs as future therapeutic targets in the pathophysiology of HF.

## Introduction

Cardiovascular diseases, heart failure (HF) included, result from numerous interactions from diverse genomic, genetic, environmental factors and lifestyle. MicroRNAs (miRNAs) display significant roles in cardiovascular pathophysiology and may even serve as diagnostic and prognostic markers for some conditions. The roles of miRNAs in signaling pathways are rapidly extending. A highly desired challenge for the function of miRNAs in HF is the possibility of using miRNAs as response assessment to therapy and even as a screening method. It is daring to hope that in the future, miRNA-mediated clinical interventions for the prevention, diagnosis, and even treatment of HF will be possible [1].

Heart failure, one of the leading causes of mortality and morbidity worldwide, is defined by the American Heart Association and American College of Cardiology as a complex clinical syndrome that can result from any structural or functional cardiac disorder that impairs the ability of the ventricle to fill with or eject blood”. Regarding signs and symptoms, the most important ones are orthopnea, paroxysmal nocturnal dyspnea, lower extremity edema, and fatigue. Based on the ejection fraction (EF) of the left ventricle, there can be patients with HF with preserved EF and patients with HF with reduced EF [1]. HF most often occurs as the last stage of decompensation of many pre-existing disorders, such as chronic high blood pressure, coronary artery disease, myocardial infarction, myocarditis, mitral regurgitation by overload, drug abuse, or as a result of chemotherapy. Some other factors such as congenital heart defects, abnormal heart valves, diabetes, sleep apnea, and alcohol abuse can also contribute [2].

Left-sided heart failure appears when the heart’s left ventricle can no longer pump enough blood; therefore, pressure and volume in the pulmonary veins increase, resulting in pulmonary congestion, shortness of breath, paroxysmal nocturnal dyspnea, orthopnea, fatigue, weakness, and reduced ability to exercise. Right-sided heart failure happens when the the right ventricle is not strong enough to pump blood into the pulmonary circulation. As a result, the high venous pressure leads to ascites, hepatosplenomegaly, dilated jugular veins, weight gain, and gastrointestinal distress [3]. miRNAs represent a new set of endogenous gene regulators, which usually include about 22 nucleotides [4], organized as single-stranded RNAs (ribonucleic acid), which shape the expression of most messenger RNAs (mRNAs) [5]. They represent a category of non-protein-coding small RNA molecules with significant roles in regulating gene expression [6] at a post-transcriptional level [4]. miRNAs are essential for a proper foundation and adequate performance of all biological processes in humans. Therefore, any disruption of miRNAs functions results in a potential pathophysiological effect [7]. miRNAs are mostly transcribed from DNA sequences into primary miRNAs, transformed into precursor miRNAs, and consequently mature miRNAs. Usually, miRNAs cooperate with the 3’ untranslated region of the target messenger RNA (mRNA), leading to mRNA degeneration and translational repression. miRNAs are also capable of triggering translation or modulating transcription [6]. miRNAs display a variety of actions, which depend on their location, their amount, target mRNAs, and compatibility of miRNA-mRNA cooperation [6].

Increasingly thought-provoking results have been done since the discovery of miRNAs in 1993 by Lee *et al.* [8]. As a consequence, one reveals for miRNAs increasing importance as diagnostic, prognostic and follow-up indicators for a multitude of conditions [9–11] and are seen as future markers for noninvasive screening [12, 13]. From the hugeness of information related to miRNAs, this review aims to point out the most important discoveries related to their usefulness in heart failure, with greater emphasis on right heart failure accompanied by hepatomegaly.

## The Biology of miRNAs

microRNAs have long been shown to control numerous biological processes, even tumorigenesis [14]. Interestingly, miRNAs are widespread in the human body, either in circulating fluids or extracellular sites [9], and play significant roles in both the normal function of systems, but also in the pathogenesis of diseases [15]. miRNAs are protected from the ribonucleic acid degradation enzyme by being included in lipoprotein complexes [9] (argonaute 2 – AGO2 [5, 16, 17], high-density lipoprotein – HDL [18–20], and nucleophosmin 1 [6, 21–23] or in extracellular vesicles [24–26] or miRNA 3’ end methylation or 3’ end nucleotide addition [9, 27]. Intracellular miRNAs are known to regulate gene differentiation, proliferation, and apoptosis [4]. Circulating miRNAs have been detected in plasma, serum [22, 28], cerebrospinal fluid [29], saliva [16], breast milk [30], urine, tears, colostrum, peritoneal fluid, bronchial lavage, seminal fluid [31], ovarian follicular fluid [32]. Circulating miRNAs are studied as potential circulating biomarkers and therapeutic targets [33] for specific diseases due to their high extracellular stability [1]. Extracellular miRNAs can also be identified in vesicles (exosomes [11, 25, 34] microvesicles and apoptotic bodies) [16, 35, 36]. Extracellular miRNAs can act as autocrine, paracrine, or endocrine controllers of cellular activities [35]. Thus, miRNAs can be considered as having hormone-like actions [6].

Furthermore, studies point to the direction of miRNAs’ roles in stem cell functions [37]. miR-1 was proved to influence skeletal and cardiac muscle development [37], miR-155 functionally inhibits the hematopoietic differentiation [38], miR-221, and miR-222 block erythropoiesis [39], miR-223 promotes granulopoiesis [40] and induces T cell lineage [37, 41]. miR-9/9, miR-22, miR-124a and miR-125b were linked to astrocyte genes suppression [42], miR-206 promotes myoblast fusion [43], miR-133 blocks mesenchymal stem cell differentiation [44], while miR-181 induces B cell differentiation [41].

## Personalized Medicine in Heart Failure

### microRNAs as diagnostic markers in HF

miRNAs can be released into extracellular fluids; therefore, they can be identified as biomarkers for a multitude of disorders [6, 45–47]. Histologically, the occurrence of HF is a consequence of tissue remodeling that occurs as the cardiovascular disease progresses. Ventricular remodeling in HF is based on numerous processes, the most important of which are cardiomyocyte hypertrophy, excessive fibrosis of interstitial tissue, decreased angiogenesis and, last but not least, apoptosis [48]. Navickas *et al.* reviewed 19 studies and aimed to determine possible miRNAs that could be used as plasma or serum biomarkers for diagnosis or prognosis for patients suffering from atherosclerosis, coronary artery disease, and acute coronary syndrome [49], concluding that miR-133a/b (5 studies), miR-208a/b (6 studies), and miR-499 (7 studies) were important markers for diagnosis and/or prognosis of cardiovascular diseases. Another conclusion was that miR-1 and miR-145b could be biomarkers for the acute coronary syndrome, while miR-1 has greater sensitivity for all types of acute myocardial infarction. miR-145 was found to be relevant for ST-segment elevation myocardial infarction and also indicates poor repercussions of acute myocardial infarction [49]. Myocardial hypertrophy represents the major adaptive process leading to HF. Studies are generally performed on animal models and have shown that there are several miRNAs, either up-regulated or down-regulated, which are involved in cardiomyocyte hypertrophy. Cardiac hypertrophic pathways were shown to be promoted by the miR-208 family, miR-212/132, miR-23, and miR-199, while miR-1, miR-133, miR-378, miR-185, and miR-155 had anti-hypertrophic effects [50–58]. What is more, miR-1, miR-133, miR-208, and miR-499 were identified to command the identity of cardiomyocytes [59]. miR-133 in inflammatory microvesicles was associated with metabolic and cardiovascular diseases [49], alongside the let-7 family, miR-17/92 family, miR-21, miR-29, miR-126, miR-133, miR-146, and miR-155 [60]. miR-126 anticipated the appearance of cardiovascular events in patients who have stable coronary artery disease [61]. Non-diabetic patients with stable coronary artery disease displayed a considerable decline of miR-126 in circulating microvesicles [62].

Cardiac fibrosis, as a consequence of the healing process, especially after myocardial infarction, leads to a hampered myocardial contractile function and represents the major cause for cardiac arrhythmias. Therefore, monitoring fibrosis is a key element, and different miRNAs involved in collagen metabolism were studied. Several pro-fibrotic ones include miR-21, miR-15, and miR-1. However, the results of many studies are controversial, at least for miR-21, which was seen as a promising drug target [63–65]. On the other hand, miR-29, miR-133, miR-26a, miR-24, miR-19a-3p/19b-3p, and miR-101a were described as antifibrotic miRNAs, being associated with the regulation of genes encoding for extracellular matrix connective tissue fibers and elements [66, 67].

The angiogenesis process is critical for myocardial repair; therefore, the maintenance of an appropriate local vascularization is the key element in preventing the hypertrophy of the working cardiomyocytes. Moreover, fibrosis accompanies reduced capillaries in tissue, and therefore, new miR-based therapies promoting angiogenesis appear nowadays as promising possibilities in the prevention of HF [68]. The primary miRNAs with anti-angiogenic function are depicted in several studies, and some can be named as more important: miR-17–92, miR-126, miR-24, miR-214, and miR-34. Others seem to induce angiogenesis, i.e., miR-210, by releasing angiogenic factors [69–71]. miR-1, along with miR-133, were identified to have roles in endothelial function and angiogenesis. miR-133 was also linked to restraining cardiac hypertrophy. miR-133, together with miR-145, can influence vascular smooth muscle cell differentiation, while miR-145 alone contributes to plaque stabilization through facilitating vascular smooth muscle and endothelial communication [49]. Furthermore, miR-17, miR-18, miR-19, and miR-20 proved to possess anti-angiogenic functions [72], while miR-92a was mostly up-regulated in ischemic conditions and inhibited angiogenesis by acting on the pro-angiogenic factor integrin α5 (ITGA5) [73].

Cardiac cell death, also known as apoptosis, also represents a key event in the progression of any cardiovascular condition towards chronic HF. The mechanism underlying this programmed cell death is still poorly understood [74]. However, several miRNAs were discovered as main contributors in the pro-apoptotic process: miR-133, miR-21, miR-30 family, miR-138, miR-499 and miR-181c [75–78]. Many of the factors listed above as being involved in the pathogenesis of HF are also involved in other processes that will ultimately contribute to the development of HF. In the review conducted by Navickas *et al.*, miR-1, miR-133, miR-499 display roles in apoptosis, while miR-1, miR-133, miR-145, miR-208, miR-499 in the differentiation of cardiac myocytes. Their part in these mechanisms could be enlightened by monitoring the shared RNA targets such as cyclin-dependent kinase inhibitor 1A (or p21), ETS proto-oncogene 1, fascin actin-bundling protein 1, hyperpolarization-activated cyclic nucleotide-gated potassium channel 4, insulin-like growth factor 1 receptor LIM and SH3 protein 1, purine nucleoside phosphorylase and transgelin 2 [49].

The overall conclusion is that plasma and/or serum levels of miR-1, miR-133a/b, miR-145, miR-208a/b, and miR-499(a) point into the direction of the most promising diagnostic features for cardiovascular diseases [49]. The important review by Navickas *et al.* revealed that miRNAs display key roles in the pathogenesis of cardiovascular diseases, supporting their applicability as diagnostic features [49]. The outcome of the study conducted by Li *et al.* was that some cardiac fibroblast-derived miRNAs (miR-660-3p, miR-665, and miR-1285-3p) were up-regulated in both heart and plasma in chronic HF, correlated with left ventricle EF and HF severity [79]. Furthermore, concentrations of circulating miR-30c and miR-181c were found to be lower in patients with chronic HF. Li and colleagues concluded that miR-660-3p, miR-665, and miR-1285-3p possess high accuracy for diagnostic purposes [79]. Concerning the relationship between miRNAs and atherosclerosis, it was suggested that while some miRNAs may play a role in atherosclerosis in certain territories, others are engaged in general mechanisms of atherosclerosis throughout the human body [47]. For example, a recurrent pattern was found in patients with carotid atherosclerosis: upregulation of miR-21 (favors angiogenesis, controls vascular smooth cell apoptosis and proliferation) and downregulation of miR-30, miR-126, and miR-221-3p. miR-21 has also been noted to be in high quantities in patients with coronary artery disease [47, 80].

Liver disease caused by HF is also known as „cardiac hepatopathy”. One of its main forms is congestive hepatopathy (CH), consisting of venous congestion associated with right-sided HF. The chain of events determined by chronic passive congestion is represented by sinusoidal hypertension, centrilobular fibrosis, cirrhosis, and even carcinogenesis (hepatocellular carcinoma) [81]. In another study, miR-122, the predominant miRNA in the liver [82], was indicated to be essential for liver homeostasis, while its loss resulted in promoting liver steatosis, inflammation, fibrosis, and even carcinogenesis [82–84]. Abu-Halima and colleagues conducted a study on liver fibrosis/cirrhosis in univentricular heart patients, with or without Fontan palliation. Univentricular heart disease (UHD) is a rare congenital heart disorder consisting of one ventricle, implying volume overload of the unique ventricular cavity and systemic venous congestion. Fontan procedure is the palliative intervention that can connect both caval veins to the pulmonary artery, conducting systemic venous blood directly into the pulmonary circulation.

Systemic venous congestion associated with this condition results in liver congestion, liver fibrosis and, later on, cirrhosis [85]. There were no specific miRNAs linked to the beginning or progression of liver fibrosis for patients with UHD. However, the study aimed to determine miRNAs that could point into the direction of important liver fibrosis in UHD patients [85]. miR-29b-3p and miR-29c-3p proved to be best related to the Model for End-stage Liver Disease (MELD)-Albumin and Albumin-Bilirubin (ALBI) scores, being in higher concentrations in patients with MELD-Albumin scores over 11 and ALBI scores over -2.6, indicating fibrosis or cirrhosis of the liver [85]. These miRNAs were also elevated in patients with UHD, especially with liver congestion and incomplete or no Fontan palliation, compared to healthy individuals. miR-29c-3p was significantly correlated with liver congestion (collapsibility index of the inferior vena cava less than 0.15), severe liver dysfunction (total bilirubin level and platelet count), and also had prognostic value [85].

Between acute HF patients and healthy individuals, low levels of miR-103, miR-142-3p, miR-30b, and miR-342-3p [86] were noted, alongside high levels of miR-499 for acute HF [87], [88]. Tijsen *et al.* found out that miR-423-5p could distinguish between healthy individuals and HF patients and also between HF and other causes of dyspnea [88, 89]]. Three studies also showed different levels of circulating miRNAs in HF with reduced ejection fraction compared to HF with preserved ejection fraction [86, 88, 90, 91]. Regarding the prognostic value of miRNAs, low levels of miR-126 were correlated with cardiovascular death in ischemic HF patients, while elevated levels of miR-508a-5p were associated with cardiovascular death in non-ischemic HF patients [92]. miR-18a-5p and miR-652-3p were decreased in HF hospitalized patients, being predictive for 180-day mortality [93]. In order for miRNAs to be used as biomarkers for the diagnosis of HF, they have to outperform natriuretic peptides or have additive value [88].

### microRNAs: Response to therapy biomarkers or therapeutic targets?

Cardiac fibrosis is a fundamental process in the evolution of HF. Zhou *et al.* pointed out that miR-503 was up-regulated in mouse heart subjects, supporting its role in the development of heart fibrosis. By promoting angiotensin II-induced fibrosis, miR-503 represents a prospective therapeutic target for decreasing cardiac fibrosis in HF patients [94]. The research conducted by Sygitowicz *et al.* pointed out that miR-29 family members (miR-29a and miR-29b), miR-150, and miR-30a-5p regulate basic processes linked to left ventricular dysfunction and heart failure after acute myocardial infarction. Moreover, these molecules could represent possible therapeutic targets during disease progression [95].

It is known that cardiac resynchronization therapy (CRT) is associated with improved performance and survival in patients with HF and electromechanical dyssynchrony. The research conducted by Marfella *et al.* included 81 patients with severe left ventricle dysfunction, significant intraventricular and interventricular dyssynchrony, and severe left ventricle dilatation. The advantages of CRT regarding left ventricle functional recovery and cardiac remodeling were linked to modulating circulating miRNAs patterns responsible for cardiomyocyte hypertrophy, fibrosis, and apoptosis for patients with HF – New York Heart Association (NYHA) classes III or IV. miRNAs associated with HF can be regulated by CRT, thus leading to favorable outcomes of heart function [96]. In animal studies, circulating levels of miR-16, miR-20b, miR-93, miR-106b, miR-223, and miR-423-5p were elevated in the subjects with hypertension-induced HF. After administering antimir for miR-208a or angiotensin-converting enzyme inhibitor, miRNAs, except for miR-19b, returned to normal levels in about eight weeks, thus proving that circulating miRNAs have a dynamic potential response to treatment [88]. In order to be sure about the possible response-to-therapy prediction, other extensive cohort studies need to be conducted. The roles of miRNAs are definitely of high interest for future therapies and follow-up in HF patients.

## Conclusion

Studies on the importance of miRNA in heart failure are becoming more numerous. Up to date, several publications describe the involvement of miRNAs in various phases of heart failure evolution. However, many issues, such as the origins of many of the circulating miRNAs, remain to be determined. Expression abnormalities of many miRNAs have been associated with different forms of heart failure, and several miRNAs were described as correlated with cardiac hypertrophy and fibrosis. Animal studies revealed that miRNAs levels and functions could be controlled both by genetic and pharmacological means, and anti-miRNA oligonucleotides can control the effects of miRNAs. The role of miRNAs as possible therapeutic targets in the treatment of heart failure emerged from ameliorated cardiac function after pharmacological interventions [97, 98]. Thereupon, it is likely that serum and/or myocardial miRNA profiling could represent biomarkers for HF severity and therapeutic response. In the future, advances in genetics and pharmacology research will hopefully lead to cardiac-specific miRNA-based therapies (miR-mimics, antagomiRs), consequently improving outcomes of HF patients.

## Acknowledgments

### Conflict of interest

The authors declare that there is no conflict of interest.
